# What Causes Seizures in Neurocysticercosis?

**DOI:** 10.1177/15357597221137418

**Published:** 2022-12-21

**Authors:** Teresa Julieta Simões Steyn, Amalia Naita Awala, Anja de Lange, Joseph Valentino Raimondo

**Affiliations:** 1 Division of Cell Biology, Department of Human Biology, Neuroscience Institute and Institute of Infectious Disease and Molecular Medicine, Faculty of Health Sciences, University of Cape Town, Western Cape, South Africa

**Keywords:** cestode, inflammation, neurocysticercosis, seizures, *Taenia solium*

## Abstract

Neurocysticercosis (NCC) is the most prevalent parasitic infection of the central nervous system. It is caused by the presence of larvae of the cestode *Taenia solium* in the brain. The most common symptom of NCC is seizures, and it is widely considered the world’s leading cause of preventable epilepsy. Despite the prevalence and impact of NCC, a thorough, mechanistic understanding of seizure generation is still lacking. In this review, we address the question “What causes seizures in NCC?” by summarizing and discussing the major theories that seek to explain the seizurogenic and epileptogenic processes in this disorder. In addition, we highlight the potential for recent advances in disease modeling to help accelerate progress in this area.

## Prevalence and Etiology of Neurocysticercosis

Neurocysticercosis (NCC) is a central nervous system (CNS) infection in humans caused by larvae of the parasitic pork tapeworm, *Taenia solium*^1^. According to the World Health Organization (2021), an estimated 2.56 to 8.30 million people globally have NCC.^2^ After lodging and developing in the human CNS, *T. solium* larvae usually remain alive for a few years, during which time the disease is usually asymptomatic.^1^ Estimates of the proportion of people with NCC who are symptomatic are challenging to come by, as asymptomatic patients are generally unlikely to undergo brain scans or serological tests that would reveal an NCC diagnosis.^3^ A few studies, have, however, attempted to determine this proportion in endemic communities, and estimates of symptomatic NCC range from 17% to 62%.^3–6^ Of those NCC patients who do have symptoms, approximately 70% to 90% experience seizures, making this the most common clinical manifestation of the disease.^3,7^ Neurocysticercosis is therefore considered the leading cause of preventable epilepsy globally.^2^ In this review, we summarize current understandings of mechanisms underlying seizures and epilepsy in NCC and outline advances in disease modeling.

## The Co-occurrence of NCC, Seizures, and Epilepsy

**Figure 1.**
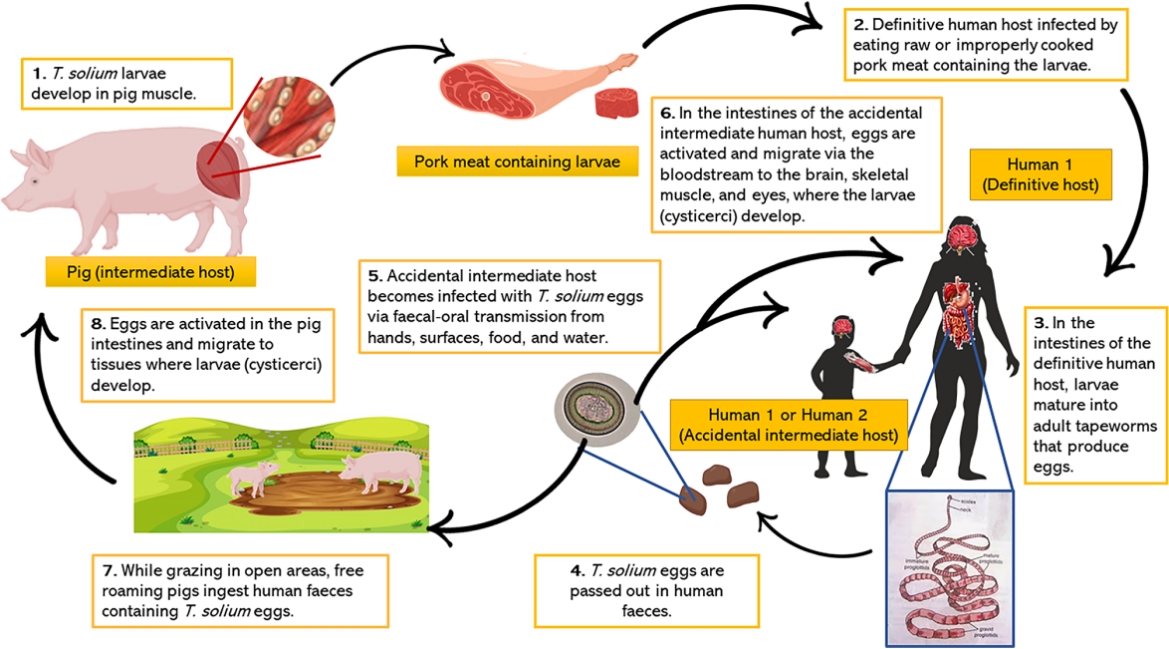
The life cycle of *Taenia solium*.

Neurocysticercosis occurs when humans become accidental intermediate hosts of *T. solium* larvae, which develop within the brain. This takes place secondary to accidental fecal-oral ingestion of *T. solium* eggs, which become activated by gastric and intestinal bile salts and enzymes and push through the gut wall into the bloodstream, eventually lodging in the CNS.^7,8^ Humans serve as definitive hosts after ingesting pork meat containing *T. solium* larvae, but not *T. solium* eggs. In this instance the host will not develop NCC, as the larvae attach to the wall of the small intestines and mature into adult tapeworms.^7,8^ Markedly, neither the adult tapeworms themselves nor the eggs they produce at this stage of the lifecycle can enter the bloodstream and cause cysticercosis, likely due to the absence of gastric elements required for their activation. See Figure 1 for an overview of the *T. solium* lifecycle and a detailed description of the above processes can be found in a recent review paper by de Lange *et al.*.^8^

In regions where NCC is endemic, which includes parts of Latin America, sub-Saharan Africa, and Asia, approximately 30% of patients with epilepsy also present with NCC infection.^9,10^ However, it should be noted that 9% to 20% of nonepileptic individuals in endemic areas also have neuroimaging and/or immunological tests compatible with NCC.^6,11–14^ Nonetheless, meta-analysis studies have found statistically significant associations between NCC infection and epilepsy, with the risk of epilepsy being estimated to be up to 3-fold higher in patients with NCC compared to people without NCC.^9,15,16^ Despite these indications, dispute still exists around whether NCC is in fact a cause of epilepsy.^9,17^ Some argue that seizure occurrence in NCC often does not fall within the strict definition of epilepsy as two or more *unprovoked* seizures occurring at least 24 hours apart,^13^ because NCC-associated seizures frequently occur secondary to an acute inflammatory response to the larval cysts, which could be considered a “provocation.”^3,9^ Therefore, discretion is urged when categorizing NCC-associated seizures as epileptic, as in some cases they may be more accurately categorized as acute symptomatic seizures even if they occur recurrently.^18–22^ Few NCC studies distinguish between acute, provoked seizures and epilepsy, making it difficult to infer the progression from “symptomatic” seizures to epilepsy in the disease.^2^ Nonetheless, some insights into the natural course of seizures in NCC have been gained from cohort studies and clinical trials. One study found that 54% of NCC patients treated with antiparasitic drugs were seizure-free after 3 years, whereas less than 2% of all untreated patients were seizure-free after this period.^23^ This implies that the majority of NCC patients with seizures will continue to experience seizures if the disease is left untreated. Furthermore, between 13% and 56% of NCC patients experience one or more seizure relapses in a period of 6 to 30 months despite receiving antiparasitic and/or antiepileptic drugs,^24–29^ suggesting relatively high rates of treatment-resistant epilepsy in NCC patients. On the other hand, these studies also provide evidence that the combination of antiparasitic and antiepileptic drugs often lead to cyst and seizure resolution, and that the progression into what can be considered epilepsy is not inevitable but instead may depend on several factors such as the number of cysts in the brain, the proportion of calcified cysts, and the presence of perilesional edema.^25,26^ Notwithstanding this issue, the high rates of comorbidity between NCC and seizures warrant controlled, experimental studies to improve understanding of the pathophysiology underlying acute and recurrent seizures in NCC.

## Possible Mechanisms Underlying Seizures in NCC

Four stages of parenchymal *T. solium* cysts have been classified according to parasite viability and pericystic host immune activity.^7^ Interestingly, these stages seem to correlate with clinical manifestations and can therefore be used to formulate hypotheses about mechanistic links between the stage of *T. solium* larval infection and seizures. The first stage, referred to as the vesicular stage, involves the presence of one or more viable cysts in the brain which appear to evade detection by the host immune system by maintaining an anti-inflammatory environment and “masking” themselves by displaying a variety of host molecules on their surfaces.^7,30–32^ Intriguingly, vesicular stage cysts are more prevalent in asymptomatic NCC patients than in symptomatic patients.^12,33^ Moreover, asymptomatic patients tend to have higher serum levels of anti-inflammatory cytokines, while symptomatic patients have been found to have higher serum levels of proinflammatory cytokines and higher levels of leukocyte adhesion molecules.^34,35^ This correlation between inflammation and symptom presentation has led to the widely held belief that it is the inflammatory response to dead or dying larvae that elicits seizures in NCC. This has been corroborated, in part, by experimental work in which intrahippocampal injections of a homogenate made from viable *Taenia crassiceps* larvae (a closely related *Taenia* species) failed to induce seizure activity in mice, while homogenates made from dying or dead granulomatous cysts did elicit seizures.^36^

Following the vesicular stage, cysts undergo the transitional stage comprising both the colloidal phase—in which cysts appear to lose their ability to control the host immune response, resulting in inflammatory cells invading the cyst wall and fluid—and the granular-nodular phase—in which the cyst cavity collapses and becomes fibrotic.^3,8^ Finally, cysts are entirely replaced with fibrotic and calcific deposits, termed the calcific stage.^3^ Notably, studies show that seizures seem to occur most frequently in patients with calcified cysts, and that these may be accompanied by pericystic inflammation, perilesional edema, and gliosis.^3,21,25,37–39^ It is also common for NCC patients to start having seizures soon after the administration of therapeutic antihelminthic drugs.^24,40,41^ This is thought to occur due to a strong inflammatory host immune response following the death of the *Taenia* larvae. Subsequently, calcified cysts and pericystic inflammation and the associated pathophysiological changes have largely been considered as the main precipitating events that lead to seizures and epilepsy in NCC. Seizures have, however, also been reported in patients with vesicular cysts and in patients with calcified cysts who lack pericystic inflammation.^21^ These cases may be explained by transient increases in the host inflammatory response that are not easily detectable,^7^ or perhaps by molecules that encourage seizurogenesis being released into the brain by the larval cysts themselves.^21^ It is highly likely that the large variability in seizure occurrence in NCC reflects multiple possible pathogenic mechanisms in the disorder, we describe these next.

Several possible mechanisms have been proposed for how inflammation can contribute to seizures. As cysts start to degenerate, they provoke a Th1 inflammatory response which is typically involved in killing intracellular parasites. Th1 responses include the release of proinflammatory cytokines and increased expression of adhesion molecules such as intercellular adhesion molecule 1 in peripheral leukocytes and in endothelial cells making up the blood–brain barrier (BBB).^35^ Upregulation of proinflammatory cytokines and adhesion molecules can influence BBB permeability, and there is indeed strong evidence that the BBB around larval cysts becomes disrupted in both mouse and pig models of NCC.^42,43^ A clinical study has also reported increased serum levels of matrix metalloproteinases in symptomatic NCC patients compared to asymptomatic patients, which is correlated with BBB breakdown.^44^ Increased BBB leakage can increase vascular permeability to serum albumin which has previously been shown (in other contexts) to facilitate epileptiform activity by compromising ion buffering and the glutamate reuptake capacity of astrocytes as well as enhancing excitatory synaptogenesis.^45–47^ Additionally, there is some evidence that certain genetic polymorphisms of inflammatory genes such as Toll-like receptor 4 (TLR4) may predispose NCC patients toward a greater risk of developing epilepsy,^48^ and this is hypothesized to be linked to TLR4’s mediation of the Th1/Th2 axis.

The notion that inflammation can contribute to seizures is well established and is supported by a breadth of literature describing the role of inflammatory molecules in exacerbating seizure activity.^49,50^ Nonetheless, we are unaware of any experimental models of seizures or epilepsy where seizure activity has been induced following an inflammatory challenge alone. Although this does not preclude inflammation as a potentially important contributor to seizure activity in certain contexts, it does suggest that, at most, inflammation is a necessary but not sufficient driver of seizure activity, and that additional factors may be required for inflammation to trigger or exacerbate seizures. Neurocysticercosis-associated seizures are no exception to this, and further research is required to truly delineate how host immune responses to *T. solium* larvae influence ictogenesis. Nevertheless, experimental animal models have provided some important insights. For example, intracranial injection of early stage *Taenia crassiceps* granuloma extracts was sufficient to induce seizures in mice and rats.^36,51,52^ This effect was found to be dependent on both the presence of substance P in the granulomas and on the host having receptors for substance P.^52,53^ Moreover, immunohistochemical analysis of brain tissue specimens from patients with NCC found substance P to be expressed in cells adjacent to remnants of dying cysts but not in regions distant from larval cysts nor in brain tissue from patients without NCC.^52^ Substance P is known to have both inflammatory and neuromodulatory properties^54^ and offers an interesting example of a molecule that could be regulating both the immune response and neuronal activity in NCC.

Perilesional edema and gliosis have also been implicated in NCC-associated epilepsy. Both processes are thought to reflect inflammatory reactions to calcified granulomas and have been strongly correlated with seizures and seizure recurrence in NCC.^38,39^ It has yet to be established, however, whether edema and gliosis are causes or consequences of seizures or whether these processes tend to coincide with seizures due to the same underlying pathophysiology.^55^ Clarity regarding the relationships between edema, gliosis, and seizures can potentially be gained from other conditions in which they co-occur, such as in traumatic brain injury and epilepsy more generally.^56–59^ Another hypothesis for how seizures arise in NCC is that calcified lesions contain and release large amounts of calcium, which could be toxic to neurons and result in high levels of glutamate being released by damaged or dying neurons, thereby facilitating seizures.^29^ Calcified cysts have also been associated with hippocampal sclerosis,^29,60^ which has a known association with medically intractable temporal lobe epilepsy that is thought to arise from structural and network changes due to neuron loss and gliosis in the hippocampus.^61^ This is another possible mechanism for epileptogenesis in NCC. Studies in which lesions associated with calcified cysts are surgically removed and seizure outcomes recorded can improve understanding about the relationship between cyst calcifications and seizures. Two such studies found that the majority of patients who received a lesionectomy for calcified cysts had favorable outcomes and became seizure-free.^62,63^ However, the small sample sizes, lack of control groups, large variability in clinical presentation of the patients undergoing these surgeries, and variability in surgery procedures leaves it unclear whether it was the removal of the calcified lesions or other factors accounting for seizure remediation in these patients.^64^ Some have argued against surgeries to remove calcified lesions as they believe there is not strong enough evidence that the benefits of such surgeries outweigh the risks,^64^ and in most cases of intraparenchymal NCC, surgeries for the removal of cysts are not indicated.^40,65,66^ Moreover, even if more robust evidence emerges demonstrating that the removal of calcified cysts improves seizure outcomes, the mechanism by which calcified cysts cause seizures will remain to be demonstrated.

A provocative idea is that some aspects of the pathophysiology of NCC-associated seizures may be comparable to seizures in patients with brain gliomas. Both *T. solium* larval cysts and gliomas are space-occupying lesions requiring a blood supply to grow and survive in the brain.^67^ Gliomas tend to develop a close association with brain vasculature leading to a breakdown of the BBB.^67^ They have also been shown to release high levels of glutamate into the surrounding tissue, which serves both as a growth factor and a neural excitotoxin to make space for the tumor’s growth.^67^ High levels of extracellular glutamate around gliomas have been shown to result in neuronal hyperexcitability in cortical networks, leading to epileptiform activity.^67^ It has also been shown that *T. solium* larvae consistently release glutamate over a period of several days when cultured *in vitro*.^68^ Based on these similarities between gliomas and *T. solium* larval cysts, it is an intriguing but untested hypothesis that similar seizure mechanisms could be at play in these two conditions.

## Recent Advances in Modeling NCC

Many of the abovementioned seizure mechanisms have been inferred from clinical correlations,^17^ rather than being experimentally demonstrated. Although various very useful model systems for investigating NCC exist, (see study by de Lange *etal.*^8^ for a detailed review) creating model systems which can accurately recapitulate important aspects of the disease including the timing, life-stage, and host–pathogen (human–cestode) interaction remains a challenge. This section will discuss recent advances in modeling NCC utilizing novel approaches.

Porcine models have long been appealing for studying disease processes in NCC as they utilize the natural intermediate host of *T. solium*. Most porcine models to date have used oral administration of *T. solium* eggs to produce cysticercosis in the pig,^69–71^ but this infection route requires an extremely high number of eggs to be administered and does not reliably result in larval infection of the brain.^63^ Recently, Arroyo et al^72^ reported on a porcine model in which activated *T. solium* eggs were administered directly into the common carotid artery, resulting in a more than 66.6% success rate of CNS cyst infection with parasitic loads that resemble that of human NCC.^73^ Although this represents a powerful new model system, it is worth noting that it is possible that some aspect of pathology in NCC results from the mismatch between the natural evolution of *Taenia* larvae to reside in pig tissue and their accidental occurrence in the human brain.

To address this, human organotypic brain slice cultures (OBSCs) could represent a useful new *in vitro* preparation for studying the pathogenesis of NCC. Rodent-derived OBSCs have long proved to be excellent model preparations for investigating a wide range of phenomena in the nervous system including synaptic plasticity, seizures, and neuroinflammation.^74^ These slices can be maintained in an incubator for up to 3 months, allowing longitudinal experimental access to brain processes, while affording the opportunity for gene transfection. The production and use of human OBSCs, using tissue collected from neurosurgical procedures, has only been described relatively recently, but it has already been used to study neurodegeneration, brain tumors, and viral infections.^75–78^ A major advantage of this model is that all resident brain cells (including innate immune cells and neurons) are retained within their native 3-dimensional tissue architecture.^75–78^ The model has certain drawbacks, however, including a lack of adaptive immunity, and the inflammatory and neurotoxic insults associated with the slicing procedure. Exposing human organotypic slices to *T. solium* larvae could provide a unique opportunity for studying pathogenic processes in NCC with unprecedented molecular and cellular resolution.

Finally, recent rapid progress in molecular biology and genetic engineering offers further novel avenues for studying the mechanisms of disease progression in NCC. The genome of *T. solium* has recently been sequenced,^79^ paving the way for analysis of different parasite variants as well as genetic manipulation of the cestode itself (eg, using CRISPR/CAS9) or creating “reporter” cestode strains. *Taenia crassiceps* larvae have, in fact, already been successfully transfected with green fluorescent protein, enabling their visualization through fluorescence microscopy.^80^ This could allow enhanced localization of the parasite as well as clearer delineation between material of host or parasite origin. Finally, next generation sequencing technologies including the latest developments in single-cell and spatial transcriptomics offer the tantalizing promise of cell-type specific and spatial readouts of both the host and parasite transcriptome at the same time.^81,82^ This is likely to revolutionize our understanding of gene expression and host–parasite interactions in NCC.

## Conclusion

Neurocysticercosis represents both a significant health challenge and a fascinating example of how multiple interacting processes between parasite and host result in the emergence of epileptic seizures. However, because the burden of disease falls largely within the developing world, relatively limited resources have been dedicated to understanding how cestode infection of the brain ultimately results in the development of epilepsy. We believe that this is a missed opportunity as advances in modeling and understanding of NCC will not only accelerate the development of much needed therapeutics for epilepsy in NCC itself but could also enable the discovery of novel antiepileptic strategies with broader applicability for treating epilepsy more generally.
